# County-scale crop yield prediction by integrating crop simulation with machine learning models

**DOI:** 10.3389/fpls.2022.1000224

**Published:** 2022-11-28

**Authors:** Saiara Samira Sajid, Mohsen Shahhosseini, Isaiah Huber, Guiping Hu, Sotirios V. Archontoulis

**Affiliations:** ^1^ Department of Industrial and Manufacturing Systems Engineering, Iowa State University, Ames, IA, United States; ^2^ Department of Agronomy, Iowa State University, Ames, IA, United States

**Keywords:** data integration, APSIM, ensemble model, spatial analysis, model transparency

## Abstract

Crop yield prediction is of great importance for decision making, yet it remains an ongoing scientific challenge. Interactions among different genetic, environmental, and management factors and uncertainty in input values are making crop yield prediction complex. Building upon a previous work in which we coupled crop modeling with machine learning (ML) models to predict maize yields for three US Corn Belt states, here, we expand the concept to the entire US Corn Belt (12 states). More specifically, we built five new ML models and their ensemble models, considering the scenarios with and without crop modeling variables. Additional input values in our models are soil, weather, management, and historical yield data. A unique aspect of our work is the spatial analysis to investigate causes for low or high model prediction errors. Our results indicated that the prediction accuracy increases by coupling crop modeling with machine learning. The ensemble model overperformed the individual ML models, having a relative root mean square error (RRMSE) of about 9% for the test years (2018, 2019, and 2020), which is comparable to previous studies. In addition, analysis of the sources of error revealed that counties and crop reporting districts with low cropland ratios have high RRMSE. Furthermore, we found that soil input data and extreme weather events were responsible for high errors in some regions. The proposed models can be deployed for large-scale prediction at the county level and, contingent upon data availability, can be utilized for field level prediction.

## 1 Introduction

Accurate crop yield prediction is necessary for agriculture production planning and the decision-making process (You et al., 2017). The crop yield depends on various factors such as genetics, environment, management, and their interactions, making it challenging to predict (Xu et al., 2019). Recent advancements in technology, data collection, and computational efficiency have facilitated the design and analysis of data-driven prediction models such as ML models (Jiang et al., 2021; Shahhosseini et al., 2021a). The accuracy, reliability, and interpretability of these models are critically important to evaluate before application in agriculture decision-making practice (Rudin, 2019).

There has been increasing interest in crop yield prediction and mainly two types of models have been designed and adopted (van Klompenburg et al., 2020). One type is based on crop simulation models, while the other type adopts a data-driven concept such as ML or Neural Networks (NN) (van Klompenburg et al., 2020). The two types of models have their own advantages and shortcomings. The crop simulation models use crop science-based formulations to capture crop physiological processes response to environmental factors, hence more explainable but difficult and costly to formulate and operate (Shahhosseini et al., 2019b). The ML models, on the other hand, once built, are easy and cheap to deploy, but the exact formulation is obscure, and the transparency can often be comprised.

ML models are designed and trained based on historical data to identify an input-output transformation function that is then utilized to predict the desired output from a set of independent variables (Singh et al., 2020). A rich body of literature concluded that ML models are very promising in predicting crop yield fields (Everingham et al., 2016; Cunha et al., 2018; Kouadio et al., 2018; Filippi et al., 2019; Shahhosseini et al., 2020; Shahhosseini et al., 2021a; Bali and Singla, 2021). In addition, to yield prediction applications, ML models had proved their efficacy in predicting nitrogen loss (Chlingaryan et al., 2018; Shahhosseini et al., 2019b) and dealing with complex biological data (Greener et al., 2021). Among the ML models, the comparative performance varies depending on the data set and response variable of interest. For example, Jeong et al. (2016) found that random forest (RF) outperformed the linear regression model in predicting grain yield for various crops (i.e., wheat, maize, and potato); similarly, Priya et al. (2018) found RF model effective for the rice crop prediction model. While Gandhi et al. (2016) used the Support Vector Machine (SVM) to build the prediction model for rice and Goldstein et al. (2018) reported that Gradient Boosted Regression Trees outperform other tree-based models in crop yield prediction. Furthermore, studies explored the ensemble of various ML models. Balakrishnan and Govindarajan Muthukumarasamy (2016) proposed two ensemble models, focusing on SVM and Naïve Bayes. Shahhosseini et al. (2020) proposed an optimized weighted ensemble model and applied it for corn yield prediction. Both studies concluded that ensemble models result in significant improvement in prediction.

Recently, crop yield prediction models based on neural networks have performed well in terms of prediction accuracy (Wang et al., 2018; Khaki and Wang, 2019; Nevavuori et al., 2019; Elavarasan and Durairaj Vincent, 2020; Shahhosseini et al., 2021b; Oikonomidis et al., 2022). Residual neural network, a combination of convolutional neural network and recursive neural network (CNN-RNN), was designed to predict corn and soybean yields across the US Corn Belt (Khaki et al., 2020). Shahhosseini et al. (2021b) designed an ensemble convolutional neural network-deep neural network (CNN-DNN) architecture to predict corn yield for 12 US Corn Belt states, which had among the lowest prediction error ever reported in the literature (RRMSE of 8.5%). A few recent studies combined probabilistic analysis with neural networks. For instance, Ma et al. (2021) developed a Bayesian neural network (BNN) to predict corn yield for the US Corn Belt. Remote sensing data are included as input variables with uncertainties considered. An average coefficient of determination (R^2^) of 0.77 was reported for the testing year from 2010 to 2019 for the U.S. Corn Belt. Abbaszadeh et al. (2022) proposed a statistical framework to perform probabilistic prediction of crop yield coupling the Bayesian model with deep learning models. It should be noted that despite the improved prediction accuracy, the underlying black-box characteristics of the neural network-based crop yield prediction models make it challenging to interpret the model and subsequent decision-making (Rudin, 2019).

Crop simulation models are pre-trained tools using field experimental data (Ahmed et al., 2016; Gaydon et al., 2017) and have been widely used by the scientific community over the past decades. In contrast to ML and NN models, crop simulation models use crop physiology, hydrology, and soil C and N cycling science-based relationships to make crop yield predictions (Asseng et al., 2014; Basso and Liu, 2019; Shahhosseini et al., 2019b), which improves the transparency and explainability. Inputs such as field management, cultivar, and daily weather are used to drive the science-based physiological relationship. For example, Togliatti et al. (2017) used the Agricultural Production Systems sIMulator (APSIM) model and forecasted weather data to predict maize and soybean yields in certain counties of Iowa (prediction error of 0.975 Mg/ha for corn and 0.608 Mg/ha for soybean), while Archontoulis et al. (2020) used the same software and historical weather records to predict corn yields for Iowa (RRMSE ~ 14%).

It has been observed in recent studies that combining ML models with crop simulation models can improve crop yield prediction accuracy and transparency. Shahhosseini et al. (2021a)found that the inclusion of outputs from crop simulation in an ML model decreased the root mean squared error (RMSE) by 8% to 20% in three US Corn Belt states (Iowa, Illinois, and Indiana). In another study, outputs from WOFOST crop growth model were combined with ML models to predict crop yield on a sub-national level for the Netherlands, Germany, and France (Paudel et al., 2021). Both studies have demonstrated significant improvement in prediction results by including outputs from a crop simulation model into ML models. However, to the best of our knowledge, no existing research work has applied this concept to other regions and at a fine spatial resolution (county level). Few researchers used neural network models to predict crop yields across the whole US Corn Belt while lacking the interpretability of the model (Khaki et al., 2020; Shahhosseini et al., 2021b).

Building upon previous research by Shahhosseini et al. (2021a) , in which they coupled ML models with crop simulation software (APSIM) to predict corn yield, here, we expand the focus of the previous research from 3 states to 12 US Corn Belt states (Illinois, Indiana, Iowa, Kansas, Michigan, Minnesota, Missouri, Nebraska, North Dakota, Ohio, South Dakota, and Wisconsin). This expansion helps to build a large-scale yield prediction model for the US Corn Belt, and the model architecture can be used to predict yields for other row crops at different scales (i.e., state, county) only by modifying the input data set. In the proposed model, APSIM outputs were combined with ML models, while the objective of the ensemble model was modified accordingly to make it suitable for mass prediction. Furthermore, for the first time, we investigate ML prediction behavior, geographic areas with low or high prediction accuracy, and the underlying factors causing it. Our specific objectives are to (a) develop a new prediction model for the entire US Corn Belt; (b) evaluate temporal prediction error at different scales: state, crop reporting district (CRD), and county (c) identify reasons for high or low prediction errors across the landscape.

## 2 Materials and methods

**Figure 1:**
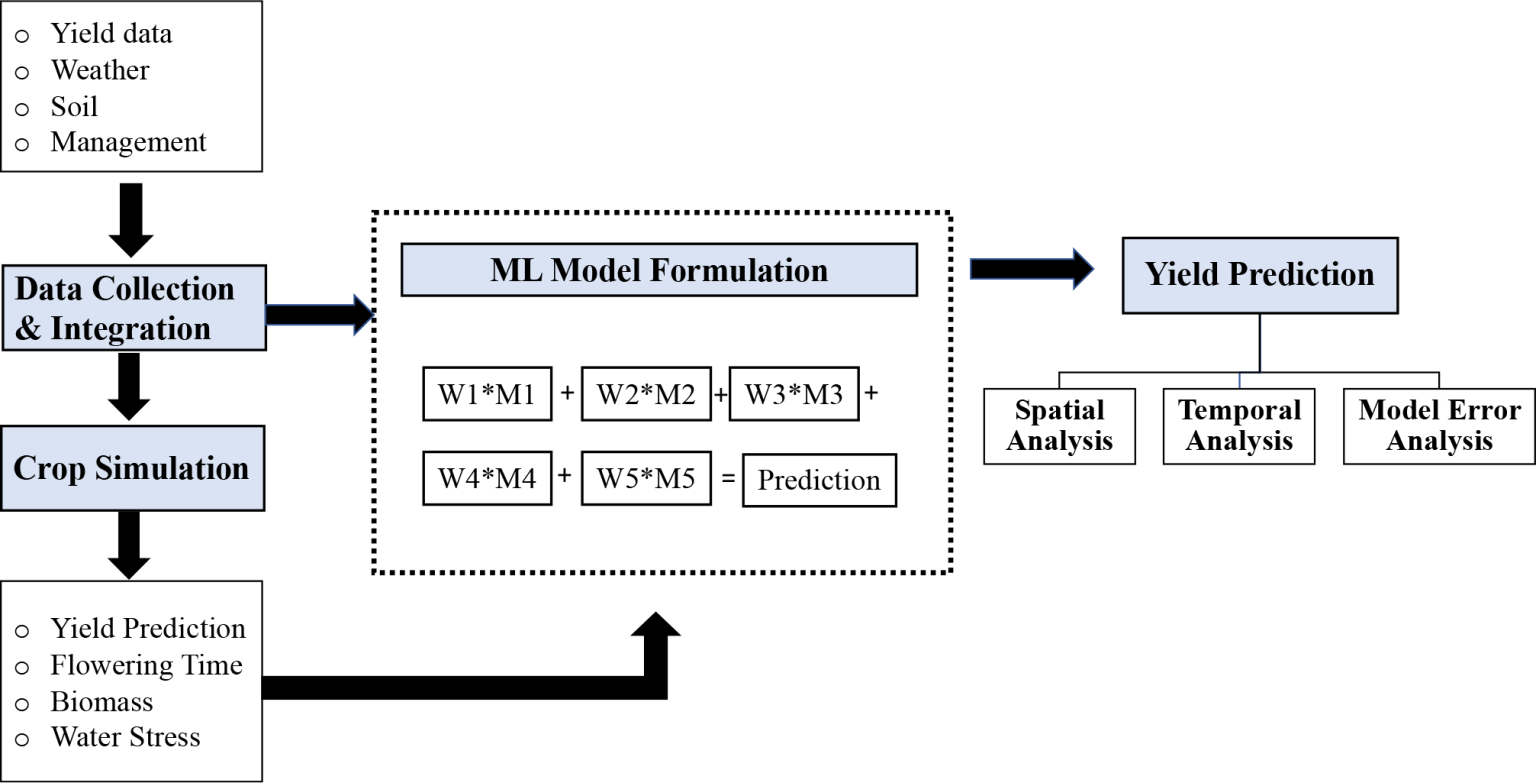
Conceptual framework of the proposed method. The first step is data collection and integration. The preprocessed data were used for both crop simulation models and ML models. The final prediction model was an optimized weighted ensemble model, where M1, M2, M3, M4, and M5 were the five prediction models, and W1, W2, W3, W4, and W5 were the weights assigned to each model.

In this study, we used publicly available soil, weather, management data (section 2.1), and simulation outputs from the APSIM model as additional inputs to the ML models (section 2.2). The research goal is to develop ML models for accurate yield prediction of the entire US Corn Belt region (sections 2.3 and 2.4). The proposed models were trained on historical data, and predictions were made for the “future” test years. In the training phase, hyperparameters of the models were optimized when building the prediction models (**Figure 1**). The prediction results were further analyzed spatially and temporally, and the source of model error was investigated.

### 2.1 Weather, soil, and management data inputs to the ML models

#### 2.1.1 Weather data

Daily weather data (radiation, precipitation, minimum and maximum temperature, and growing degree days) was obtained from Iowa Environmental Mesonet (IEM) (Iowa Environmental Mesonet) for the entire U.S. Corn Belt (1984 to 2020) and aggregated to weekly time periods for use in ML modeling (n=293 features). The weather dataset is a reanalysis product from the IEM, a more detailed description is offered in section 2.2 and in Archontoulis (2020).

#### 2.1.2 Soil data

The soil data were retrieved from SSURGO (USDA NRCS - Natural Resources Conservation Service | NRCS Soils, 2019). We used ten soil properties at ten different soil profile depths. The soil properties are soil organic matter, sand content, clay content, soil pH, soil bulk density, wilting point, field capacity, saturation point, and saturated hydraulic conductivity.

#### 2.1.3 Crop management data

Crop management data, such as planting progress data, were retrieved from NASS (NASS - Quick Stats | Ag Data Commons, 2020). This dataset contains weekly planting information at the state level for the US Corn Belt. Since there are, on average, 52 weeks in a year, this dataset has 52 features. These 52 features explain the state-wise weekly cumulative percentage of corn being planted.

#### 2.1.4 Crop yields data

The historical yield data for 12 states of the US Corn Belt were retrieved from NASS (NASS - Quick Stats | Ag Data Commons, 2020). This dataset contains annual yield data at the county level for the US Corn Belt along with the county code to specify each location.

### 2.2 APSIM model-simulated data inputs to the ML models

The APSIM (Holzworth et al., 2014) is an advanced simulator of cropping systems with many crop models along with soil water, carbon, and nitrogen models. The modules interact on a daily time step with each other. In this project, we used the APSIM maize version 7.9 for the US Corn Belt (Archontoulis et al., 2020) which is used for Iowa State extension programing, the Forecast, and Assessment of Cropping Systems project (Archontoulis and Licht, 2021). This version includes a simulation of shallow water tables and inhibition of root growth due to excess water stress (Ebrahimi-Mollabashi et al., 2019) and waterlogging functions (Pasley et al., 2020).

**Figure 2:**
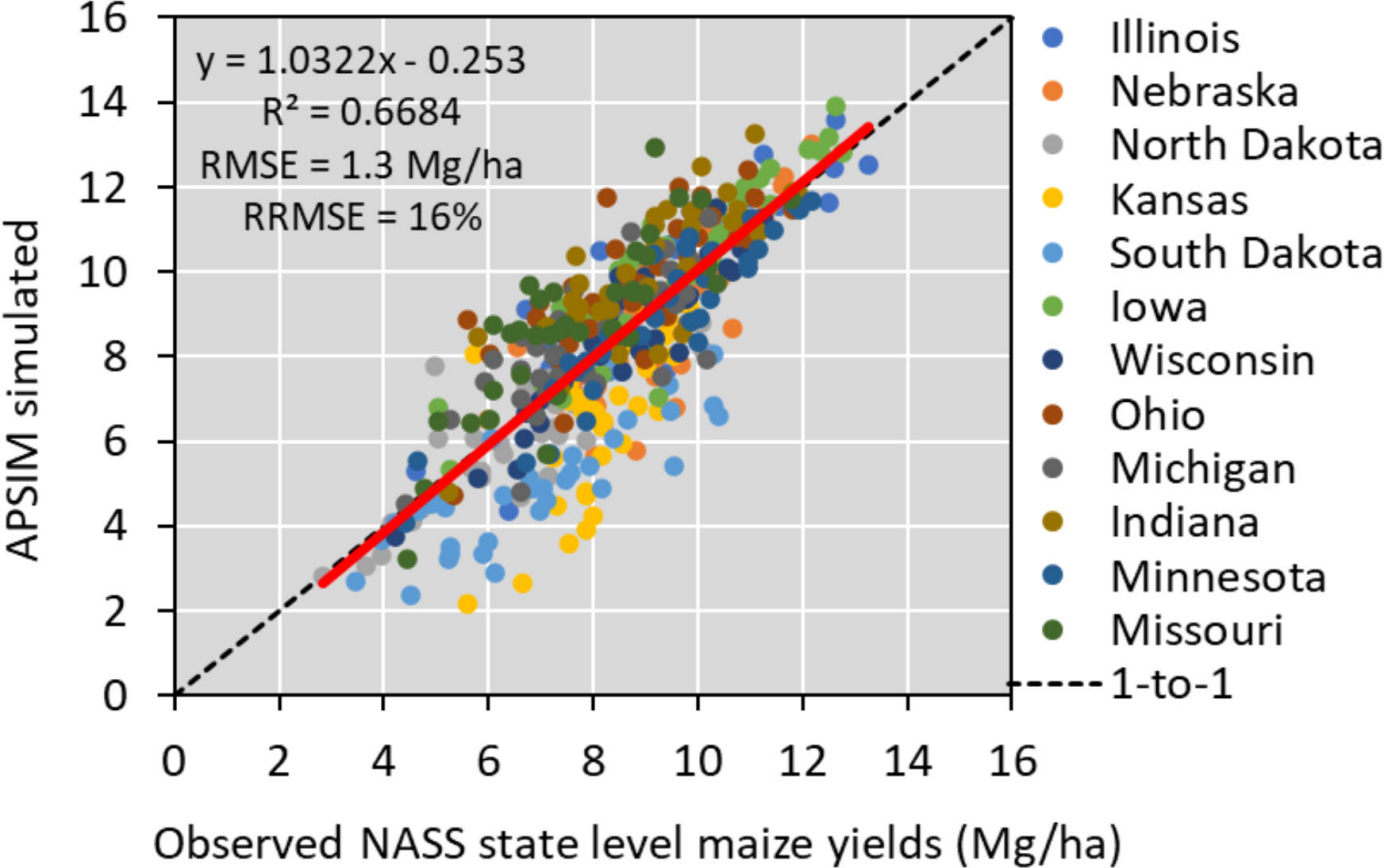
APSIM model performance in simulating historical yields in the US Corn Belt. The red line represents the relation between the predicted and observed yield for each state and has a R^2^ value of around 0.67.

To run APSIM across the 12 states, we used the parallel system for integrating impact models and sectors (pSIMS) software (Elliott et al., 2014). The simulations used in this study were created on a 5-arcminute grid across 12 states (Iowa, Illinois, Indiana, Kansas, Michigan, Minnesota, Missouri, Nebraska, North Dakota, Ohio, South Dakota, and Wisconsin), considering only cropland areas when creating soil profiles. To operate the model, we used the same source of weather, soil, and management data described in section 2.1. Soil data from SSURGO (Soil Survey Staff et al., 2020), weather data from Iowa Environmental Mesonet (mesonet.agron.iastate.edu). and management input databases (plant density and planting date by year and by state) from (NASS - Quick Stats | Ag Data Commons, 2020). In the APSIM model, cultivar traits data were derived through regional-scale model calibration. N fertilizer data was derived from a combined analysis of USDA-NASS (NASS - Quick Stats | Ag Data Commons, 2020) and Cao et al. (2018), including N rates for corn by county and by year. Over the historical period, 1984–2020, APSIM captured 67% of the variability in the NASS crop yields having an RMSE of 1.3 Mg/ha and RRMSE of 16% (**Figure 2**). This version of the model was used to provide outputs to train the ML algorithm (**Table 1**).

**Table 1:** APSIM model simulated variables considered as input features in the ML models.

Acronym	Description
AnnualYield	Crop yield (kg/ha)
AnnualBiomass	Above ground crop biomass at maturity (kg/ha)
AnnualRootD	Maximum root depth (mm)
DOY_Flowering	Flowering time (doy)
DOY_Maturity	Maturity time (doy)
AnnualLaiMax	Maximum leaf area index (m2/m2)
AnnualET	Actual evapotranspiration (mm)
MaizeTranspiration	Crop transpiration (mm)
AnnualNupt	Crop n uptake (kg n/ha)
AnnualGrainlNupt	Grain N uptake (kg N/ha)
AvgDroughtStress	Average drought stress on leaf development (0–1)
AvgExcessWStress	Average excess moisture stress on photosynthesis (0–1)
AvgNStress	Average N stress on grain growth (0–1)
AnnualAvgWT	Depth to water table during the entire year (mm)
AnnualRunoff	Runoff (mm)
AnnualDrainage	Drainage from tiles and below 1.5 m (mm)
AnnualGrossMiner	Soil gross N mineralization (kg N/ha)
AnnualNlossTotal	Total N loss (denitrification and leaching) (kg N/ha)
DOY_Sowing	Sowing time (day of year)
DOY_Harvest	Harvesting time (day of year)
SW1m_Apr	Soil water ratio at 0-1m depth for April
SW1m_May	Soil water ratio at 0-1m depth for May
SW1m_Jun	Soil water ratio at 0-1m depth for June
SW1m_Jul	Soil water ratio at 0-1m depth for July
SW1m_Aug	Soil water ratio at 0-1m depth for August
SW1m_Sep	Soil water ratio at 0-1m depth for September
SW1m_Oct	Soil water ratio at 0-1m depth for October
SW15cm_Apr	Soil water ratio at 0-15cm depth for April
SW15cm_May	Soil water ratio at 0-15cm depth for May
SW15cm_Jun	Soil water ratio at 0-15cm depth for June
SW15cm_Jul	Soil water ratio at 0-15cm depth for July
SW15cm_Aug	Soil water ratio at 0-15cm depth for August
SW15cm_Sep	Soil water ratio at 0-15cm depth for September
SW15cm_Oct	Soil water ratio at 0-15cm depth for October
WTatPlanting	Water table depth at planting
SW45_excess	Growing season average excess soil water stress
SW45_deficit	Growing season average drought water stress

Shahhosseini et al. (2021a) found that APSIM simulated soil water variables to be one of the important features for ML yield prediction. Given the extended focus of this study, from 3 to 12 states compared to Shahhosseini et al. (2021a), we used more features of the APSIM to re-evaluate the previous finding.

### 2.3 Data preprocessing

Prior to developing ML prediction models, we conducted the following data processing. First, data from all sources were aggregated while treating any missing values. Thereafter, new features were constructed to improve the model’s performance. After feature construction, a set of important features was identified and used to develop the final prediction models.

#### 2.3.1 Data aggregation and imputing missing values

Data obtained from all sources were aggregated, ensuring the proper order according to year, state, and county. The data from different sources were aggregated based on the unique GEOID (county-scale) and year associated with each row of data. Since the soil data did not vary over the year, the soil data were merged based on the unique GEOID. In this task, the historical yield data obtained from NASS (NASS - Quick Stats | Ag Data Commons, 2020) was considered as a reference point while merging data from various sources.

Afterward, the missing values were treated. The missing values were mostly appearing in the planting progress data for North Dakota State, as the values were available from the year 2000. Whereas for this paper, the data were collected from the year 1984 to 2020. The planting progress data contains the planting information on a weekly level. The planting progress of week “*n*” from 1984 to 1999 was imputed manually by the average of the planting progress data of North Dakota from the year 2000 to 2020 for a week “*n.”* Utilizing a similar approach, planting progress for the weeks of interest was imputed. The granularity level of APSIM and UDSA county-level data had some discrepancies, which caused some missing values in APSIM data. These missing values are imputed by the median value at the county level. Imputing with median values aids in the reduction of outliers (Shahhosseini et al., 2021a).

#### 2.3.2 Feature construction

##### 2.3.2.1 Weather information

The weather data contained weekly weather information, and from these weekly data, new features for quarterly data were generated. For precipitation, solar radiation, and growing degree days, a summation of daily data was considered to create the quarterly data. While for minimum and maximum temperature, the average of quarterly data was utilized to generate the quarterly minimum and maximum temperature.

##### 2.3.2.1 Yield trend

An improvement in corn yield was observed over the years, resulting from advancements in genetics and management practices. To include this yield trend in our ML model, a new feature yield trend was constructed at the county level.


(1) $$y_{i}=\;b_{0}+b_{1}x_{i}$$

*Where*, *y_i_* = Yield trend of year *x* at location *i*

*x_i_* = Year of location *i*

#### 2.3.3 Feature selection

The combined data set with information on weather, soil, management practices, and features from APSIM resulted in a large number of input variables (n= 550). Using all these features in the prediction model will make the prediction model inclined to overfitting. Thus, feature selection is essential to ensure that the designed ML model is generic. In this paper, we reduced the input features from 550 to 100 by using a two-stage feature selection approach. The first stage is based on expert knowledge, and the second stage is based on the permutation feature selection approach.

##### 2.3.3.1 Expert knowledge-based feature selection

For weather features, we considered data from weeks 16 to 43 by removing weather features at the end of the harvesting season until a few weeks before planting the next crop. Similar considerations were made for planting progress data by selecting cumulative planting progress from weeks 12 to 29, as data beyond this window does not include valuable information. All 37 APSIM features were kept intact in this step, and they were selected based on the APSIM features found important by Shahhosseini et al. (2021a). Using the expert opinion from 550 features, it was reduced to 298 features.

##### 2.3.3.2 Permutation feature selection

In the second stage of feature selection, permutation-based feature selection with the random forest was used. This approach can overcome bias in default random forest variable importance, and importance is calculated based on the impurity (Strobl et al., 2007; Altmann et al., 2010). Furthermore, the feature importance algorithm of the random forest provides consistent feature ranking for high dimensional feature space with multicollinearity (Maciej et al., 2013). In permutation feature importance, the importance of an input variable is computed by a change in the model’s error with and without the feature. In the validation set, the values of the feature are shuffled, and if this results in an increase in model error, the feature is considered important (Breiman, 2001; Molnar, 2020). This process is repeated until all features are ranked based on their importance.

For permutation-based feature selection, a ML model must be fit. In this research, a random forest model with 300 trees and other default parameters was fitted to the permutation feature selector. The numbers of trees used in the random forest were tuned through the grid-search method with ten-fold cross-validation. Finally, the top 100 features were selected and were further considered as input to the prediction model.

### 2.4 Prediction models

Selecting diversified and individually credible base learners are crucial for high-performing ensemble models (Brown, 2017). Hence, different ML models were developed, and their individual and combined performances were compared to identify the best-performing model. The base models applied in this study are linear regression, LASSO regression, random forest, XGBoost, and LightGBM, which had proved their prediction efficacy in earlier studies (Shahhosseini et al., 2020; Shahhosseini et al., 2021a). Furthermore, to have a well-performing model, tuning the hyper-parameters of these base models is vital. Grid search is widely used to tune hyperparameters of ML models. However, hyper-parameter tuning through grid search is a cumbersome task because it evaluates the model performance for each combination of the hyper-parameters. The Bayesian search method can overcome this challenge. In the Bayesian search approach, an underlying distribution of model parameters is assumed, and this belief (prior) is updated along with new observations. Bayesian optimization aims to balance exploration (gathering more information on hyper-parameters) and exploitation (decision based on available information) while collecting the maximum amount of information (Snoek et al., 2012). Therefore, to tune the hyper-parameters Bayesian search with 40 iterations with a 10-fold cross-validation procedure was adopted for model formulation.

#### 2.4.1 Linear regression

Linear regression tries to identify the linear relationship between the variables and response variables. It assumes that residuals have a normal distribution with constant variance and that predictors do not have any correlation (James et al., 2013). In this paper, we used a multiple linear regression model.

#### 2.4.2 LASSO regression

LASSO is an L1-regularization model. It has a built-in feature selection design, where it assigns zero to the coefficient of features with less importance (Tibshiranit, 1996; James et al., 2013). The loss function, which is mean squared error (MSE), has an additional penalty term that ensures only the important features are selected for the model formulation (Tibshiranit, 1996).

#### 2.4.3 Random forest

In a random forest, multiple data sets are created based on the bootstrap resampling method, which is sampling with replacement (Breiman, 2001). Each sample set and a subset of features are used to build trees, and this process is repeated (Brown, 2017). The final prediction is made from the majority vote by aggregating those trees. As each tree is uncorrelated, random forest balances both bias and variance components in the loss function (Cutler et al., 2007).

#### 2.4.4 XGBoost and LightGBM

The concept of gradient boosting tree-based model is incorporated in both XGBoost and LightGBM. Weak tree-based models are sequentially built-in gradient boosting using the knowledge of the prior weak model. In contrast to the random forest in gradient boosting, the final prediction is the accumulation of sequentially built models. XGBoost was proposed by Chen and Guestrin (2016), and LightGBM is an updated version proposed with better computational power by Microsoft (Ke et al., 2017).

#### 2.4.5 Average weighted ensemble

In the ensemble model, several base learners are merged together, and the final prediction is a combination of predictions from all models. The final prediction made by the ensemble model has better accuracy than the base learners (Grossman et al., 2010). In an average weighted ensemble, equal weights are given to each prediction model. The final prediction is made based on this weighted average value. However, to have a better average weighted ensemble model, it is necessary to use diversified models as based models (Brown, 2017).

#### 2.4.6 Optimized weighted ensemble

Compared to the weighted average ensemble model, the optimized weighted ensemble model selects weights considering the base model performance in the training set, providing a higher weight to better-performing learners and improving the model accuracy. Shahhosseini et al. (2019a) reported that an optimized weighted model improves the model performance compared to the average weighted ensemble model. The optimum weight was determined by minimizing the mean squared error (MSE) of training data. For large-scale data, using the minimization of MSE as an objective-function result in assigning the majority weight to a single model. Since MSE is the square of observations, for large-scale data, MSE from each model has a higher value, making the objective value less sensitive to the weight given to each model. To address the concern, the objective function is modified to select weights by minimizing the RMSE of the training set instead of MSE. This modification retains the objective function in the same order of observation.

**Decision variable**

*w_j_* is the weight associated with each base model *j* (1, 2, …. K)

**Parameters**

*y_i_* is the *i*th observation of the response variable 

${\hat{y}}_{ij}$ is the prediction of *i*th observation by *j*th model


(2) $$\begin{array}{l} Objective:\\ \;\min\sqrt{\frac{1}{n}{\sum_{i=1}^{n}\left(y_{i}-\sum_{j=1}^{k}w_{j}{\hat{y}}_{ij}\right)}^{2}} \end{array}$$

(3) $$\begin{array}{l} s.t.\\ \;\sum_{j=1}^{k}w_{j}=1 \end{array}$$

(4) $$\sqrt{\frac{1}{n}\sum_{i=1}^{n}{\left(y_{i}-\sum_{j=1}^{k}w_{j}{\hat{y}}_{ij}\right)}^{2}}-\sqrt{\frac{1}{n}\sum_{i=1}^{n}{\left(y_{i}-{\hat{y}}_{ij}\right)}^{2}}\leq 0\;\forall j=1,\;2......k$$

(5) $$w_{j}\geq 0,\;\forall j=1,2......k$$

The optimum weights are determined by solving the minimization model (eqs 2-4). The objective function aims at selecting the weights such that the RMSE of the entire ensemble model is minimized with the constraint that all weights must sum up to 1(eq 3). In addition, the weights of the final model are selected in a way that the ensemble model has lower or equal RMSE of any base learner (eq 4), ensuring that the optimized ensemble model improves the model performance.

For all base models and ensemble model outputs from APSIM, weather, soil data, and management practices from 1984 to a year before (*‘n-1’*) the prediction year (*‘n’*) was given as an input. For example, if predicting 2020, data from 1984 to 2019 was used to train the model.

### 2.5 Evaluation metrics

All the ML models trained for the period 1984 to 2017 and their prediction capacity (R^2^) and error (RMSE, MBE) were evaluated in the years 2018, 2019, and 2020 for all the US Corn Belt (Illinois, Indiana, Iowa, Kansas, Michigan, Minnesota, Missouri, Nebraska, North Dakota, Ohio, South Dakota, and Wisconsin). In 2020, the derecho that occurred in Iowa reduced crop yield (Derecho | IOWA HSEMD). The 27 counties severely impacted by derecho were disregarded for the 2020 yield database. To evaluate model performance, we used 4 statistical evaluation metrics (eqs 6-9) and residual plots between observed and simulated data.

*Root Mean Squared Error (RMSE)*, which is the square root of the mean squared error (MSE), that measures the average squared difference between the predicted and the observed values (the lower the value the better):


(6) $$RMSE=\;\sqrt{\frac{\sum_{i=1}^{n}{\left(y_{i}-{\hat{y}}_{i}\right)}^{2}}{n}}$$

*Relative Root Mean Squared Error (RRMSE)*, which is normalized RMSE, values ranging from 0-100% (the lower the better). In this paper, the value is normalized by dividing RMSE by the mean of observed values:


(7) $$RRMSE=\;\frac{RMSE}{\frac{\sum_{i=1}^{n}y_{i}}{n}}$$

*Mean Bias Error (MBE)*, which identifies the bias of the prediction model, the average deviation of predicted values from the observed values (the lower the value the better):


(8) $$MBE=\;\frac{\sum_{i=1}^{n}\left({\hat{y}}_{i}-y_{i}\right)}{n}$$

*R^2^ score*, which is known as the coefficient of determination. It provides information on the model’s ability to capture the variability in data. The value of R^2^ ranges from 0 to 1, a higher value indicating that the model performs well:


(9) $$R^{2}=1-\frac{RSS\left(sum\;of\;square\;of\;residuals\right)}{TSS\;\left(Total\;sum\;of\;sqaures\right)}=1-\frac{\sum_{i=1}^{n}{\left(y_{i}-{\hat{y}}_{i}\right)}^{2}}{\sum_{i=1}^{n}{\left(y_{i}-\bar{y}\right)}^{2}}$$

After determining the overall best performing model, the results were analyzed to have interpretability. This analysis focus on identifying some key reasons for variation in model performance across different time frame and geographical locations. Any relation between the source of error and weather and soil properties was assessed state-wise. To perform this analysis, the Pearson correlation coefficient between the RMSE and various model features was calculated.

## 3 Results

### 3.1 Model performance

The developed ML models had RMSE from 0.99 to 1.45 (Mg/ha), RRMSE from 8 to 14%, R^2^ from 0.5 to 0.75, and MBE from -0.78 to 0.03 (Mg/ha) across all test years and locations (**Table 2**). The optimized weighted ensemble outperformed all the prediction ML models, with RRMSE from 9 to 9.35% and R2 from 0.72 to 0.8.

**Table 2:** Model evaluation statistical values.

Criteria	Year	Model						
		LASSO	XGBoost	LightGBM	Random Forest	Linear Regression	Optimized Weighted Ensemble	Average Weighted Ensemble
RMSE (Mg/ha)	2018	1.43	1.04	1.01	1.08	1.45	1.01	1.14
	2019	0.96	0.92	0.94	0.94	1.1	0.92	0.91
	2020	1.11	0.99	1	1.12	1.12	0.99	1.01
RRMSE (%)	2018	13.25	9.61	9.35	10.05	13.42	9.35	10.54
	2019	9.37	8.98	9.22	9.22	10.75	9.22	8.87
	2020	10.26	8.84	9	10.04	10.05	9	9.07
R2-Score (0-1)	2018	0.59	0.78	0.8	0.76	0.58	0.8	0.74
	2019	0.71	0.73	0.72	0.72	0.62	0.72	0.74
	2020	0.67	0.75	0.75	0.68	0.68	0.75	0.74
MBE (Mg/ha)	2018	-0.78	-0.16	-0.16	-0.4	-0.62	-0.16	-0.43
	2019	-0.32	-0.16	-0.24	-0.06	-0.5	-0.24	-0.26
	2020	-0.48	-0.13	-0.06	-0.14	0.03	-0.06	-0.16

**Figure 3:**
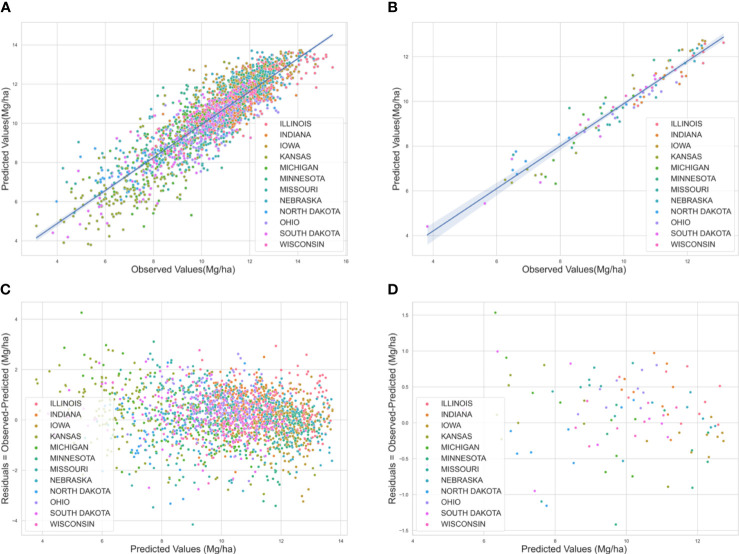
**(A)** Predicted versus observed yield for the U.S Corn Belt for the years 2018-2020 at the county level. The straight lines at 45^0^ show a strong correlation between the predicted and observed yield **(B)** at the crop reporting district level. **(C)** Residual plot for the U.S Corn Belt for the years 2018-2020 at the county level **(D)** residual plot at the crop reporting district level. The errors are scattered around zero lines, indicating an unbiased model. Each color corresponds to a particular state.

Graphical analysis of measured versus predicted values revealed a weaker correlation for low maize yields whereas, a stronger linear correlation for higher yield regions (**Figure 3**). The ML predictions were more reliable for locations with normal to high yields compared to regions with lower yields. These areas mostly belong to Kansas and South Dakota which had an average low cropland area (5-20% cropland per state).

In addition, the robustness of the developed prediction model was evaluated by exploring residual plots (**Figure 3**). The errors in the residual plots from the model for the years 2018, 2019, and 2020 were random and well distributed for both the county level and for crop reporting districts. This indicates that the model had good performance in terms of being unbiased.

After analyzing the performance of all the models using different evaluation criteria (**Table 2**, **Figure 3**) we found that the optimized ensemble model outperformed all other models; therefore, this model was considered to be the final model, and the prediction results from this model were used for further analysis.

### 3.2 Spatial and temporal analyses of model performance

**Figure 4:**
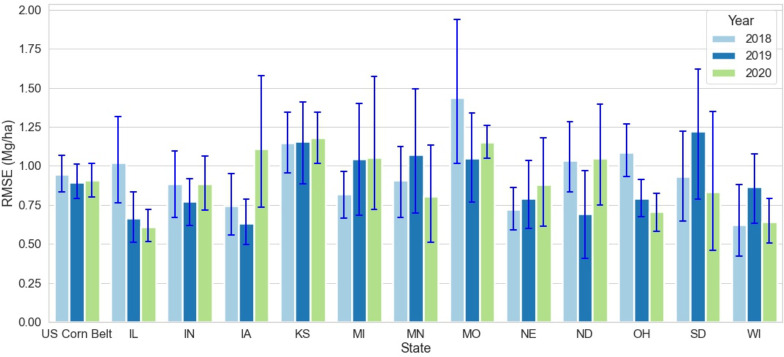
State-wise RMSE for optimized weighted ensemble model from the year 2018 to 2020. The blue lines demonstrate the range of RMSE for each state at the crop reporting district level for each year. MO had the highest RMSE in 2018 IL had the lowest RMSE in 2020.

Across 3 test years and 12 states, the RMSE of the optimized weighted ensemble model varied from 0.6 to 1.4 (Mg/Ha) (**Figure 4**). The highest RMSE was observed in IL, OH, and MO in the year 2018; and in IA in the year 2020. It should be noted that the high RMSE for IA in 2020 was because of derecho (Derecho | IOWA HSEMD). Even though the 27 counties severely impacted by derecho were disregarded for analysis, derecho caused an overall reduction in corn yield for Iowa in 2020, making a significant deviation from the yield trend and causing higher error for Iowa in 2020.

**Figure 5:**
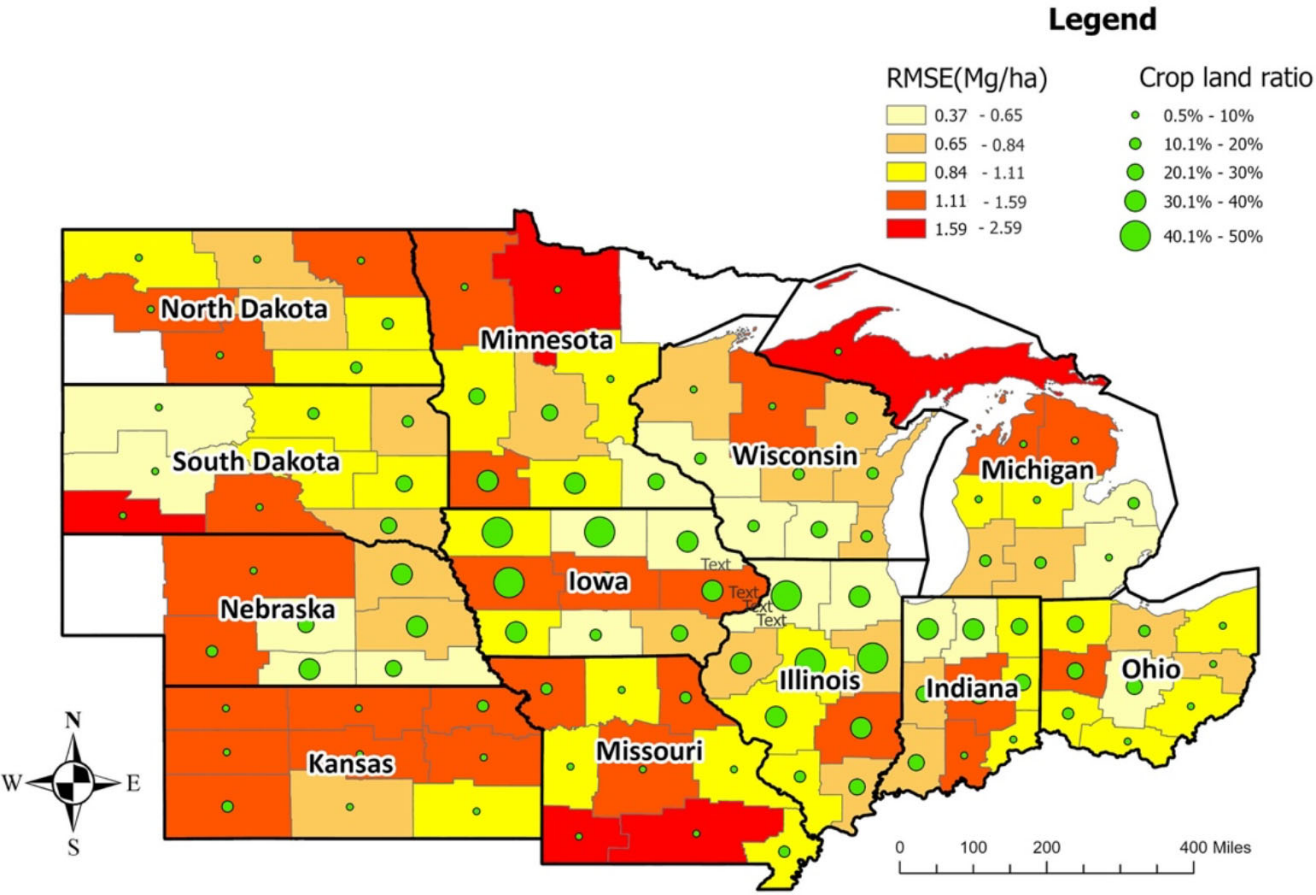
Average model performance at CRD level in terms of RMSE (the year 2018-2020). The lighter color corresponds to lower RMSE (ranging from 0.37 to 1.11Mg/Ha), whereas the darker color stands for higher RMSE (ranging from 1.11 to 2.59 Mg/Ha). The green circles provide information on the cropland ratio of the crop reporting districts. Overall, Iowa, Illinois, Indiana, and Ohio have lower prediction errors.

The crop reporting districts, mostly from Iowa, Illinois, Indiana, and Wisconsin, had the lowest RMSE, while the Upper Peninsula crop reporting district from Michigan had the highest RMSE (**Figure 5**). The crop reporting districts with a higher cropland ratio had relatively lower RMSE, indicating that the prediction model is more reliable for areas with a higher cropland ratio as more data is available for those locations (**Figure 5**, **Supplementary Figures 1-3**). An exception was Central Iowa, even with a higher cropland ratio, which had a higher average RMSE (deriving from the RMSE of Central Iowa in 2020, in which derecho impacted crop yields).

**Figure 6:**
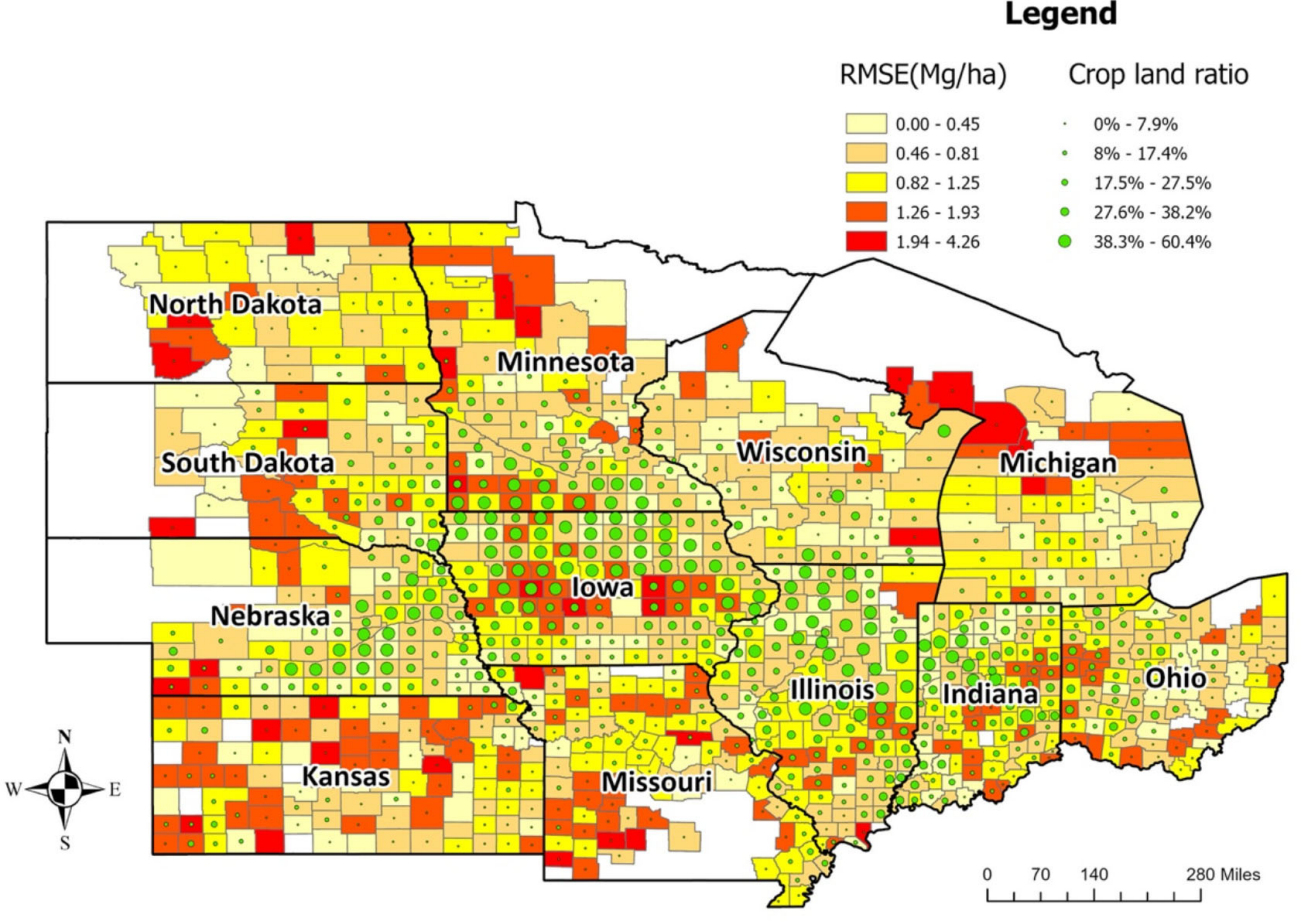
Average model performance at the county level in terms of RMSE (the year 2018-2020). The lighter color corresponds to lower RMSE (ranging from 0 to 1.25Mg/Ha), whereas the darker color stands for higher RMSE (ranging from 1.26 to 4.26Mg/Ha). The green circles provide information on the cropland ratio of the county. Iowa, Illinois, Indiana, Ohio, and Nebraska have lower prediction errors.

The averaged RMSE of the optimized ensemble model at the county level for the years 2018, 2019, and 2020 were lower in counties from Iowa, Illinois, Indiana, Ohio, and Wisconsin (**Figure 6**, **Supplementary Figures 4-6**). The relationship between model performance (RMSE) and cropland ratio at the county level was found to be similar to the relationship at the CRD level. Counties with higher cropland ratios had lower errors, which means the model is more reliable in high-yield areas.

### 3.3 Relationship between model performance and input features

**Figure 7:**
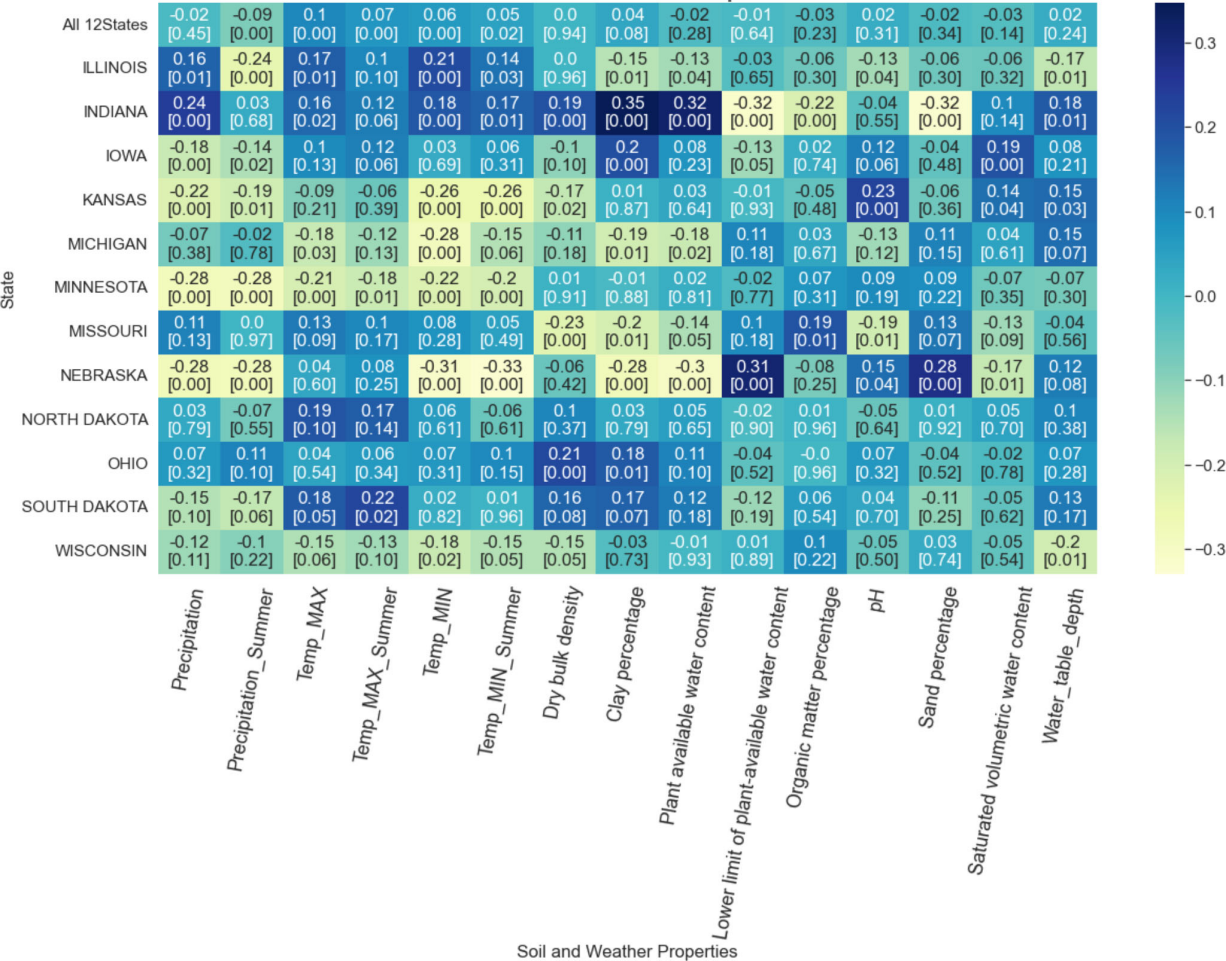
Correlation between soil-weather properties and RMSE. The darker the color, the higher the linear correlation between the RMSE of the model and the feature along the x-axis. The first row provides the overall correlation for all 12 states with the feature along the x-axis. The remaining rows demonstrate the correlation at the state level. The first value in each box represents the correlation value, and the second value corresponds to the associated p-value.

The Pearson correlation coefficient between soil-weather properties and RMSE ranged from -0.33 to 0.35, indicating no strong correlation between soil-weather properties and RMSE (**Figure 7**). For certain States, some features are negatively correlated to RMSE, whereas, in other states, they have a positive correlation. Across the soil-weather variables, the RMSE is mostly correlated with maximum and minimum temperature (annual and summer), followed by soil properties clay percentage (**Figure 7**).

The correlation between weather properties and RMSE varied from -0.32 to 0.24 (**Figure 7**). Both annual and summer precipitation had a mixed impact on RMSE. For instance, higher annual precipitation in Indiana increases RMSE, while higher summer precipitation relates to a lower RMSE. No consistent pattern was observed with high or low RMSE and precipitation regimes. For Nebraska, the RMSE was mostly correlated with rain.

Similarly, an association of RMSE with maximum and minimum temperature was random. However, it should be noted that the direction of correlation for annual and summer temperature was the same, both for maximum and minimum temperature. In North Dakota, higher maximum annual or summer temperature was related to higher RMSE, while in Nebraska, higher minimum annual or summer temperature was related to lower RMSE. In North Dakota, higher maximum annual or summer temperature was related to higher RMSE (p-value > 0.05), while in Nebraska, higher minimum annual or summer temperature was related to lower RMSE. The associated p-value indicates that the correlation with all-weather variables for Minnesota is significant (p-value< 0.05), while the correlations were insignificant for Missouri, North Dakota, and Ohio. Correlation with specific weather features was significant for other states; for instance, Iowa, Kansas, and Nebraska correlated significantly with precipitation and minimum temperature.

A similar random relationship was found for soil properties and RMSE, Pearson correlation coefficient varying from -0.32 to 0.35 for different states (**Figure 7**). For Indiana, a higher clay percentage was related to a higher model RMSE (p-value<0.005). At the same time, high RMSE in Nebraska was related to low plant-available water content and clay percentage and with high sand percentage and lower limit of plant-available water content (p-value<0.005). For Missouri, high RMSE was related to high organic matter percentage (p-value = 0.01). In fact, high pH was related to high RMSE for Kansas (p-value<0.005); the low sand percentage was correlated to high RMSE for Nebraska (p-value<0.005), and high saturated volumetric water content was related to higher RMSE for Iowa (p-value<0.005). High water table depth was related to low RMSE for Illinois and Wisconsin (p-value = 0.01); in contrast, it was related to high RMSE for Indiana (p-value = 0.01) and Kansas (p-value = 0.03). Finally, observing the correlation with RMSE and soil-water properties for all 12 states, it can be concluded that the source of error is random, with no strong correlation with the certain soil-water property (all p-value > 0.05).

## 4 Discussion

Based on the comprehensive analysis of the evaluation matrices, the optimized ensemble model was the best-performing model for maize yield prediction in the US Corn Belt (**Table 2**). In a few cases, for example, in 2019, the RMSE & RRMSE of the average weighted ensemble model are moderately better than the optimized weighted ensemble model. The reason is that one base model LASSO had relatively poor performance for training years while performing well in the test year 2019. As the optimized weighted ensemble model assigns weights to a base model considering the performance in training years, it assigns a low weight to LASSO for predicting the test year. Though the average weighted ensemble performed marginally better in terms of RMSE and RRMSE for 2019, it had a higher bias than the optimized weighted ensemble model.

In comparison, the optimized weighted ensemble model has a consistent performance in terms of all different evaluation matrices for three consecutive years for 12-states. Hence, the optimized ensemble model results were selected for further temporal and spatial analyses. For reference, in crop modeling research, several investigators found that the multi-model average provides the best prediction of crop yields (Asseng et al., 2014; Iizumi et al., 2018; Heino et al., 2020; Shin et al., 2020; Shahhosseini et al., 2021a). It appears that the use of multi-model is a viable way toward increasing prediction in agriculture at the expense of additional model runs and time. Further analysis of the model’s sensitivity revealed that extreme weather conditions did not extensively impact model performance. The model performance was tested against varying weather properties, and the model was able to maintain a certain accuracy level.

The inputs of the prediction models play a vital role in model performance. Among different sets of factors, weather (temperature, rainfall, humidity, solar radiation, precipitation), and soil information (soil type, soil maps, soil pH-value), images were widely used in previous studies (van Klompenburg et al., 2020). Along with weather data, information on soil properties improves the performance of the prediction model (Dai et al., 2011). Leuthold et al. (2022) applied a linear regression model to identify the correlation between topography and corn (crop) yield, where the relationship varied with precipitation. Some recent studies focused on understanding the impact of soil properties and meteorology on crop yield (Guo et al., 2021; Jiang et al., 2021). Weather, soil, and management information were fed as input to the ML models and focused on interpreting the model performance with different weather and soil conditions. However, by evaluating the correlation between weather and soil properties, it was found that the source of error in the model is random (**Figure 7**). Using choropleth maps, we also spatially analyzed the model performance at the county level (**Figure 6**) and for different crop reporting districts (**Figure 5**). This analysis revealed that the model performs better in locations with a higher cropland ratio.

**Figure 8:**
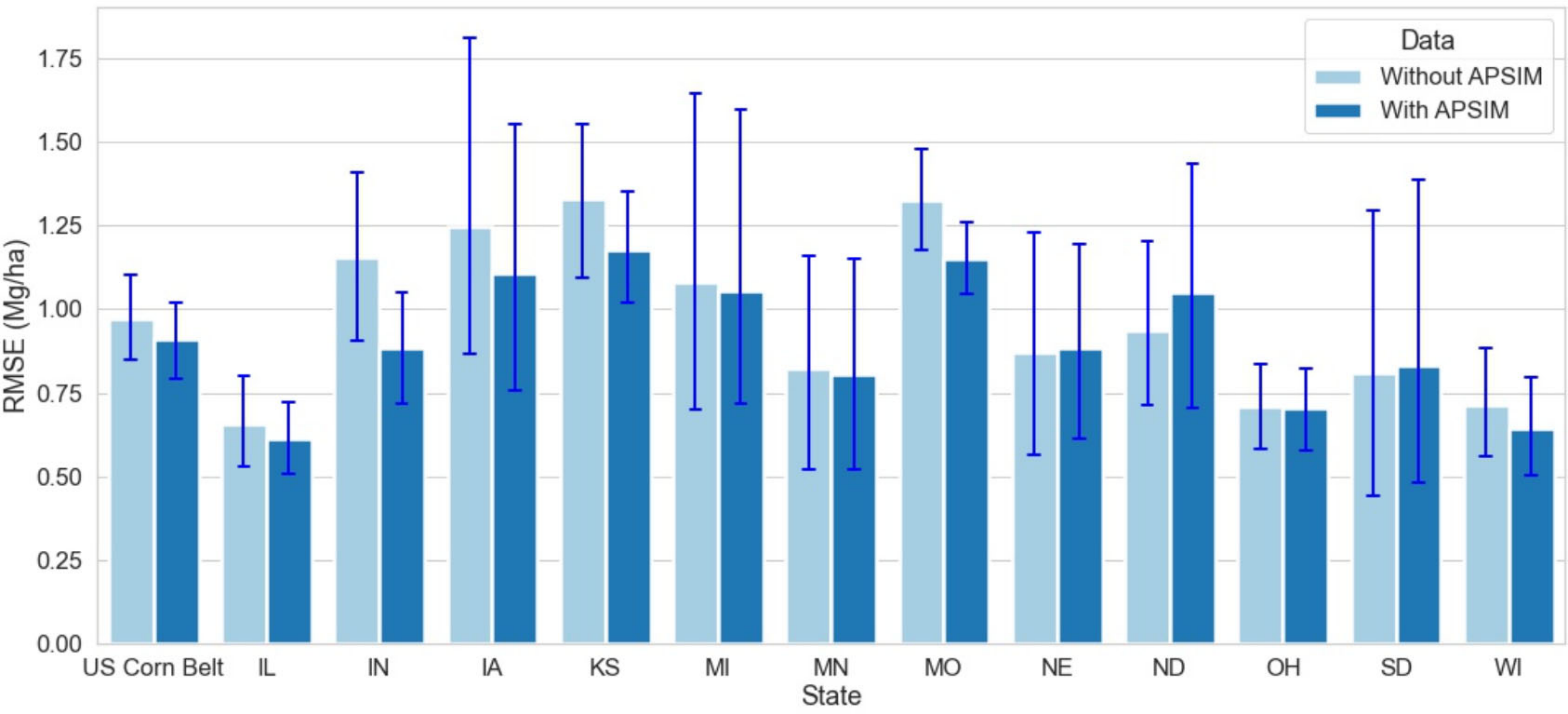
Model Performance with and without APSIM for the year 2020 for 12 states of the US Corn Belt. The blue lines demonstrate the range of RMSE with and without APSIM. The first set of bars compares the model performance for the US Corn Belt, while the other set of bars compares the model performance at the state level.

This study builds upon Shahhosseini et al. (2021a) work, where it was reported that coupling ML with crop simulation software (APSIM) improves the model performance. Shahhosseini et al. (2021a) applied the coupled model to three states, Indiana, Illinois, and Iowa, in 2018 and found an RRMSE of 7.5%. This study expands the analysis to twelve US Corn Belt states and reveals that for all states except North Dakota, the coupled ML model with APSIM data outperforms the model without APSIM (**Figure 8**). Thus, coupling crop modeling with ML is a way towards increasing prediction accuracy in agriculture.

While developing the prediction model for 12 states, we found that minimizing MSE to determine optimum weights for base models in the ensemble fails to assign optimum weights. Using minimization of MSE assigns all weight to the best-performing model in the training set, which impairs the benefits of the ensemble model. The objective function was modified to address this challenge and build more that can be applied on a large scale. In our proposed ensemble model, instead of minimizing MSE, we minimized the RMSE of the model. This modification resolves the issue. To verify the consistency in performance for the proposed prediction model, results were compared for similar years and states, as Shahhosseini et al. (2021a) reported. It was observed that the proposed model has an RMSE of 1.025 Mg/ha for those three states. For the year 2018, the mean yield for these three states was 12.18 Mg/ha, which results in an RRMSE of 7.8% for the prediction model. This comparison suggests that even after 12 states, the proposed model can maintain its consistency in performance.

**Figure 9:**
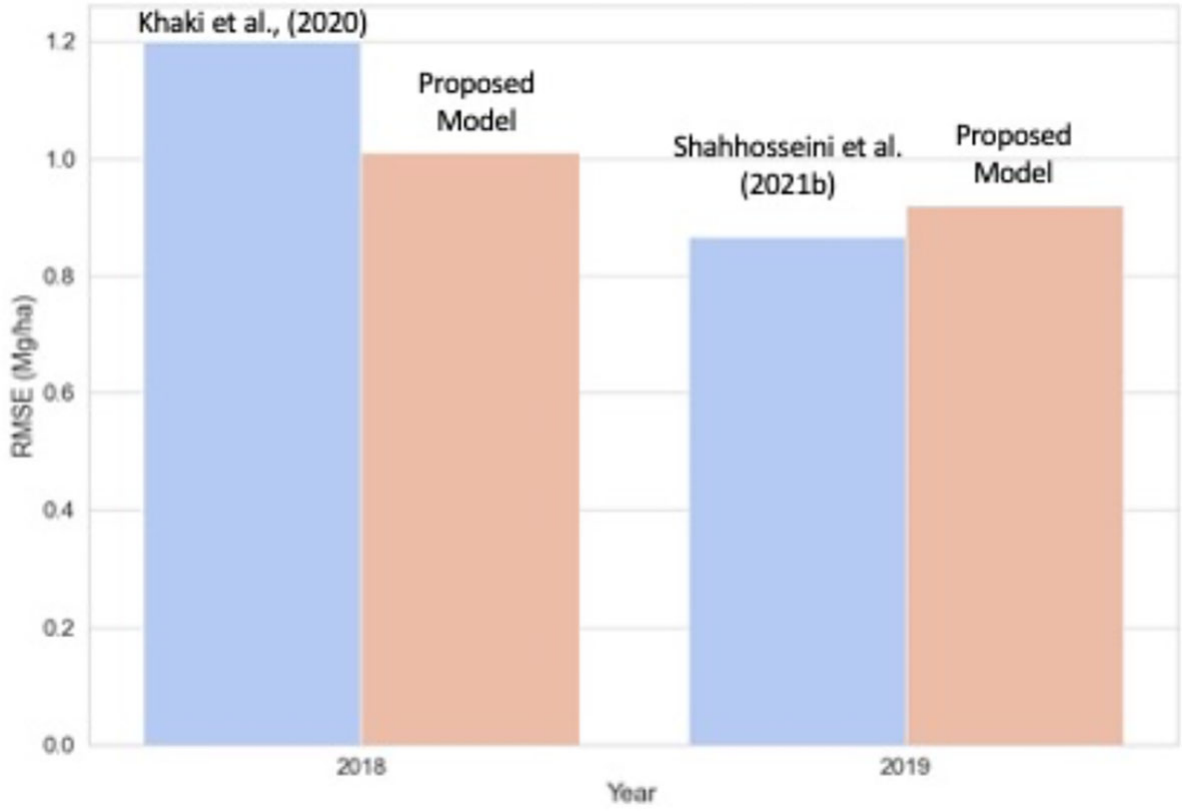
Comparison of the proposed model with state-of-art corn yield prediction model for the US Corn Belt. For 2018, the proposed model is compared against Khaki et al. (2020), and for the year 2019, the model is with Shahhosseini et al. (2021b).

We further compared the proposed model performance with two state-of-art models based on neural networks (**Figure 9**). Khaki et al. (2020) achieved an RMSE of 1.2 Mg/ha for predicting corn yield for the US Corn Belt for 2018. Our proposed new model for the same year (2018) had a similar RMSE (1.1 Mg/ha) while providing better interpretability. Shahhosseini et al. (2021b) predicted corn yield for the same 12-states in 2019 by implementing an ensemble CNN-DNN model with the weather, soil, and management data. For 2019, the obtained RRMSE was around 8.5%, which is similar to our work (average ensemble model RRMSE of 8.8% and optimized weighted ensemble model RRMSE of 8.9%). Hence, the proposed model can maintain similar accuracy as the CNN-DNN model while with additional benefits in terms of interpretability.

The model performance presented in **Figure 5** and **Figure 6** is based on RMSE. However, to decide on the reliability of a model, one single criterion is insufficient. Another model evaluation criterion is RRMSE. In RRMSE, RMSE is divided by the mean of the corresponding observations. This approach can create inconsistency in evaluating a model for a different period. The mean yield for the 12 states for 2018, 2019, and 2020 were 10.77, 10.23, and 10.42 Mg/ha, respectively. When models are evaluated temporally, a model with the same RMSE will have a lower RRMSE in 2018, whereas a higher RRMSE in 2020. This approach may cause rejecting a reliable model in one year whereas accepting it in another year. So, this will cause complications in selecting a model which performs well generically.

This study developed a robust modeling system to predict county-scale yields. Future studies can leverage this system and climate change scenarios to inform policy, aiding decision-making. In addition, RMSE, RRMSE, and other evaluation criteria can be explored to find an evolution matrix that can ensure the identification of a reliable model on a generic level. Furthermore, data from remote sensors can be included as an input feature to develop better-performing models.

## 5 Conclusion

This research developed a crop yield prediction model for the US Corn Belt that aims to be applicable for a wide region and with improved model transparency. We integrated the outputs from the crop growth model (APSIM) together will soil, weather, and management data into ML models to build the crop yield prediction model. The ensemble of various ML models was found to be the best-performing model. Analysis of model performance temporally and on different geographic levels (county and crop reporting districts) revealed that areas with a higher cropland ratio have a lower model prediction error. Our study is among the first to explain the reasons for the low/high prediction accuracy of ML models. Finally, we confirmed that coupling crop modeling with ML increases crop yield predictability in larger geographic areas than previously reported. For future research, the focus can be improving the model performance for locations with limited historical data by incorporating remote sensing data.

## Data availability statement

The original contributions presented in the study are included in the article/**Supplementary Material**. Further inquiries can be directed to the corresponding author.

## Author contributions

SS conducted the research and wrote the manuscript. GH oversaw the research and edited the manuscript. MS contributed to the research idea and data processing. SA provided the data and guidance for the research and edited the manuscript. IH prepared the APSIM data. All authors contributed to the article and approved the submitted version.

## Funding

This work was partially supported by the National Science Foundation under the LEAP HI and GOALI programs (Grant number 1830478). This work was also supported by the Plant Sciences Institute’s Faculty Scholars program at Iowa State University.

## Conflict of interest

The authors declare that the research was conducted in the absence of any commercial or financial relationships that could be construed as a potential conflict of interest.

## Publisher’s note

All claims expressed in this article are solely those of the authors and do not necessarily represent those of their affiliated organizations, or those of the publisher, the editors and the reviewers. Any product that may be evaluated in this article, or claim that may be made by its manufacturer, is not guaranteed or endorsed by the publisher.
