# Supplementary material for: County-scale crop yield prediction by integrating crop simulation with machine learning models
Source: Front Plant Sci. 2022 Nov 28;13:1000224. doi: 10.3389/fpls.2022.1000224 (PMC9742473; doi:10.3389/fpls.2022.1000224)
Supplement: Supplementary file 1 [file DataSheet_1.docx]

**
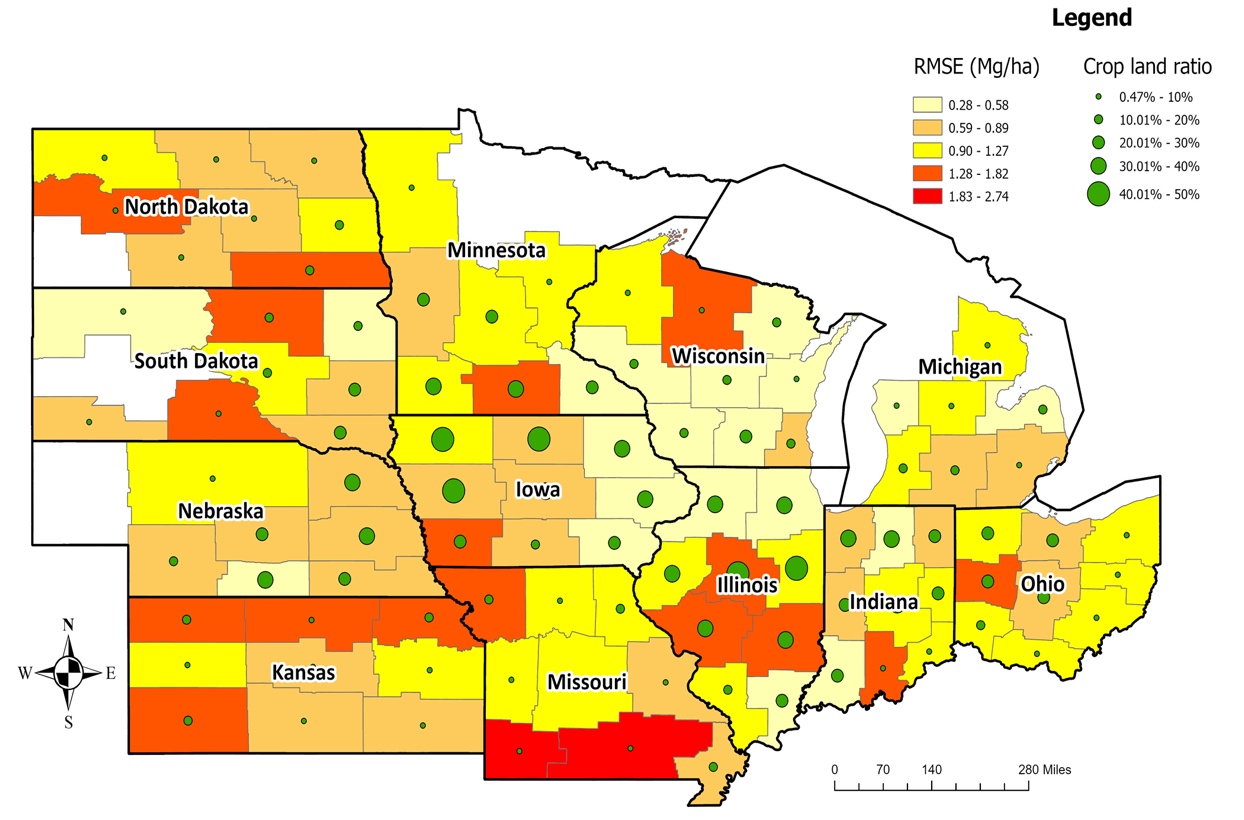
**

**Supplementary Figure 1.** Model performance at CRD level in terms of RMSE for the year 2018. The lighter color corresponds to lower RMSE, whereas the darker color stands for higher RMSE. The green circles provide information on the cropland ratio of the crop reporting districts.

**
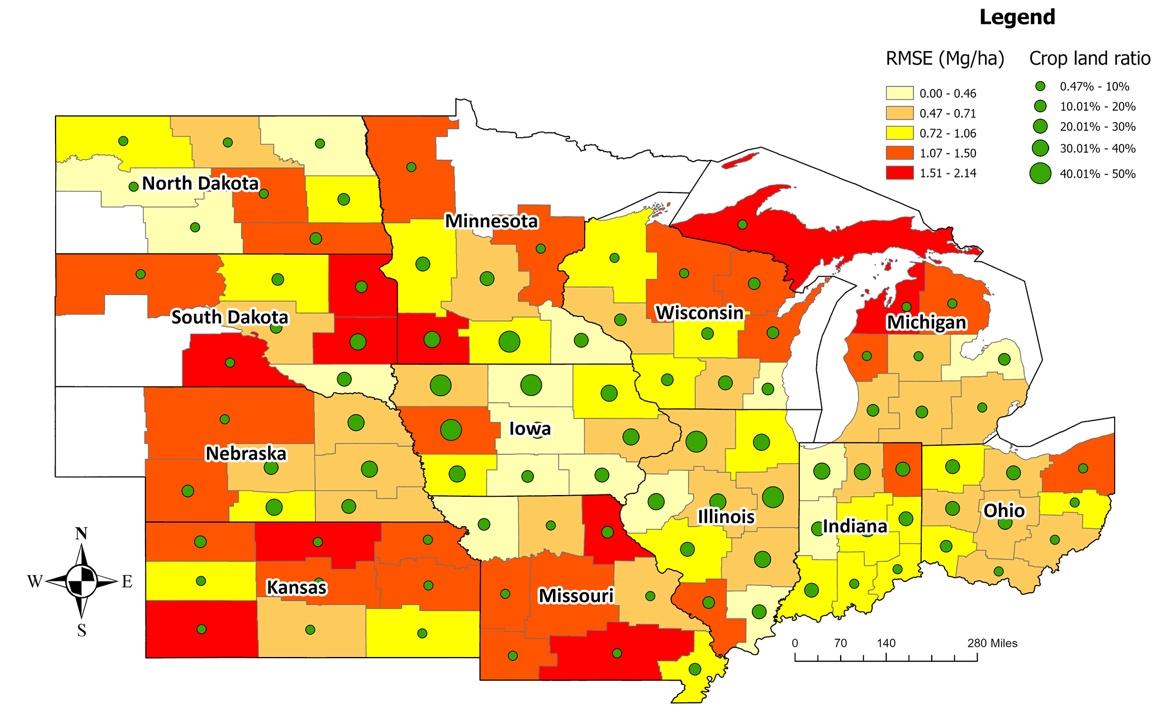
**

**Supplementary Figure 2.** Model performance at CRD level in terms of RMSE for the year 2019. The lighter color corresponds to lower RMSE, whereas the darker color stands for higher RMSE. The green circles provide information on the cropland ratio of the crop reporting districts.

**
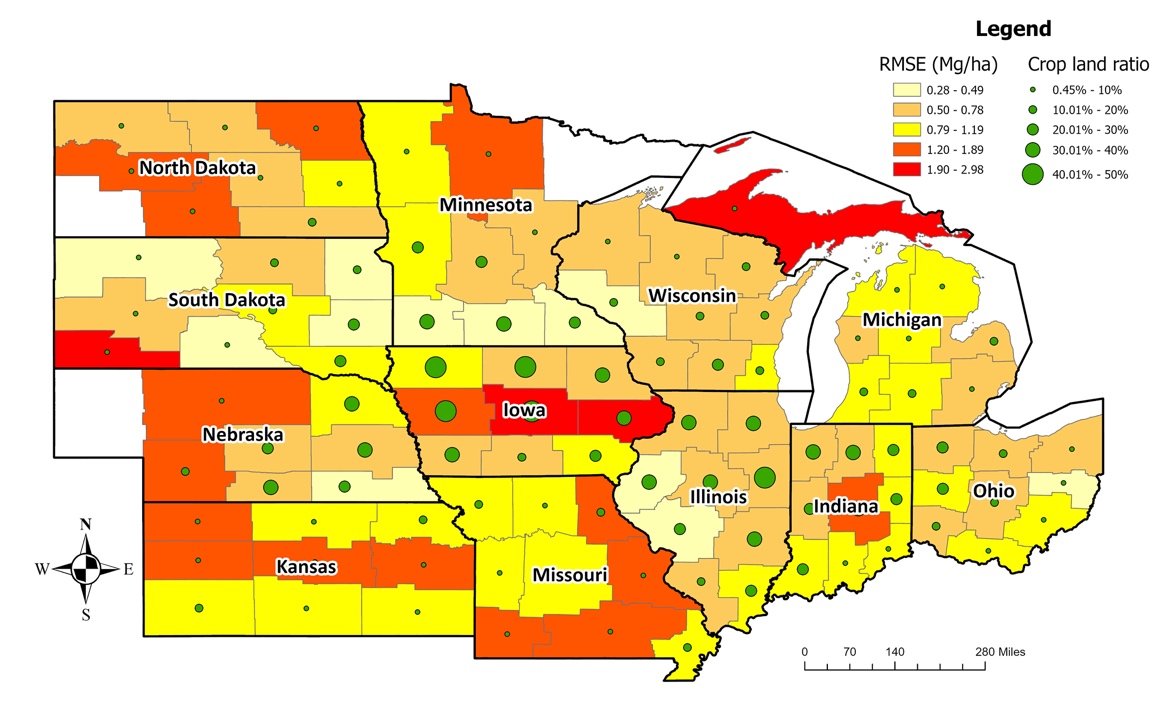
**

**Supplementary Figure 3.** Model performance at CRD level in terms of RMSE for the year 2020. The lighter color corresponds to lower RMSE, whereas the darker color stands for higher RMSE. The green circles provide information on the cropland ratio of the crop reporting districts.

**
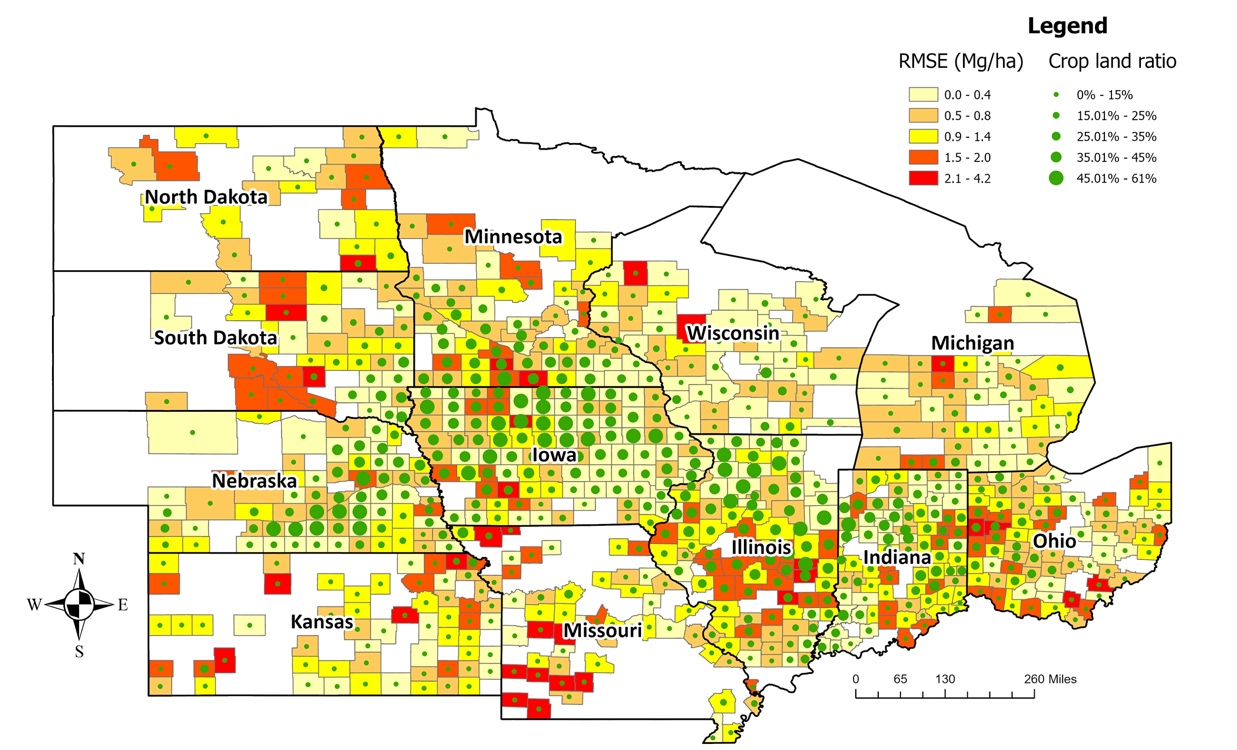
**

**Supplementary Figure 4.** Model performance at the county level in terms of RMSE for the year 2018. The lighter color corresponds to lower RMSE, whereas the darker color stands for higher RMSE. The green circles provide information on the cropland ratio of the county.

**
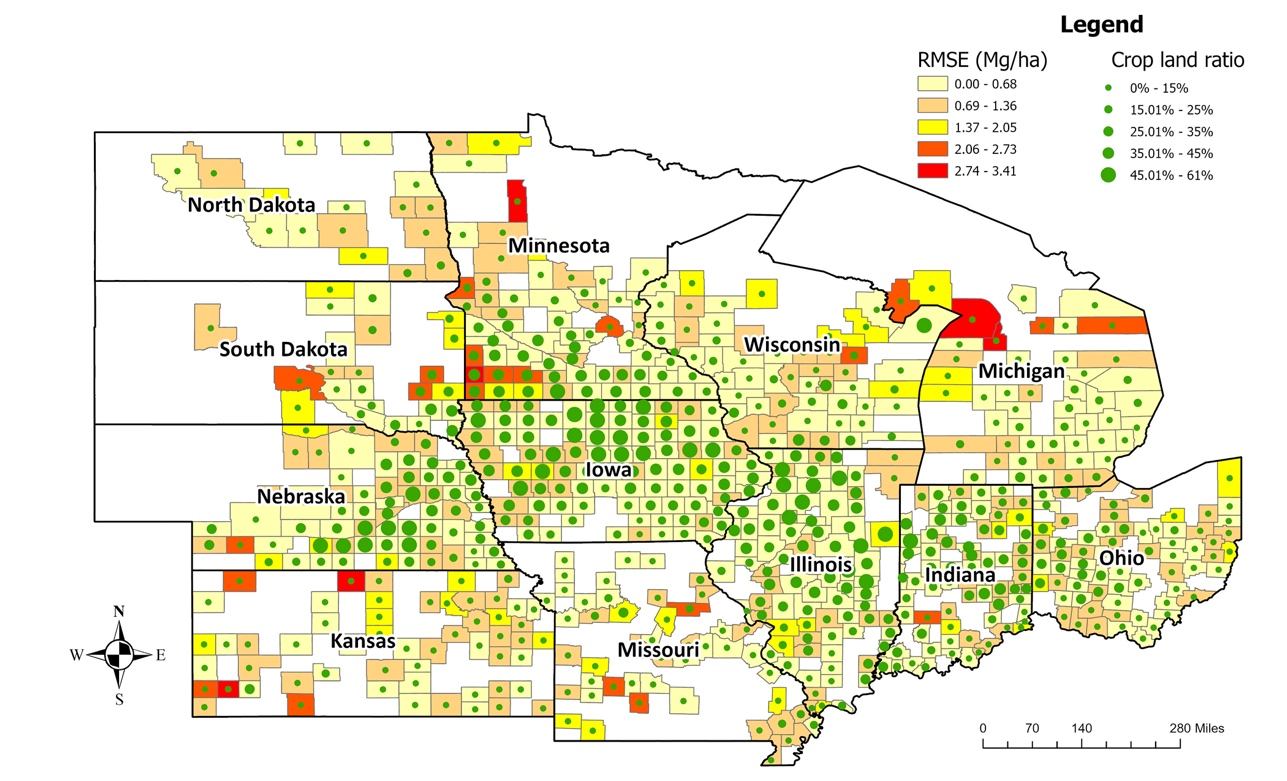
**

**Supplementary Figure 5.** Model performance at the county level in terms of RMSE for the year 2019. The lighter color corresponds to lower RMSE, whereas the darker color stands for higher RMSE. The green circles provide information on the cropland ratio of the county.

**
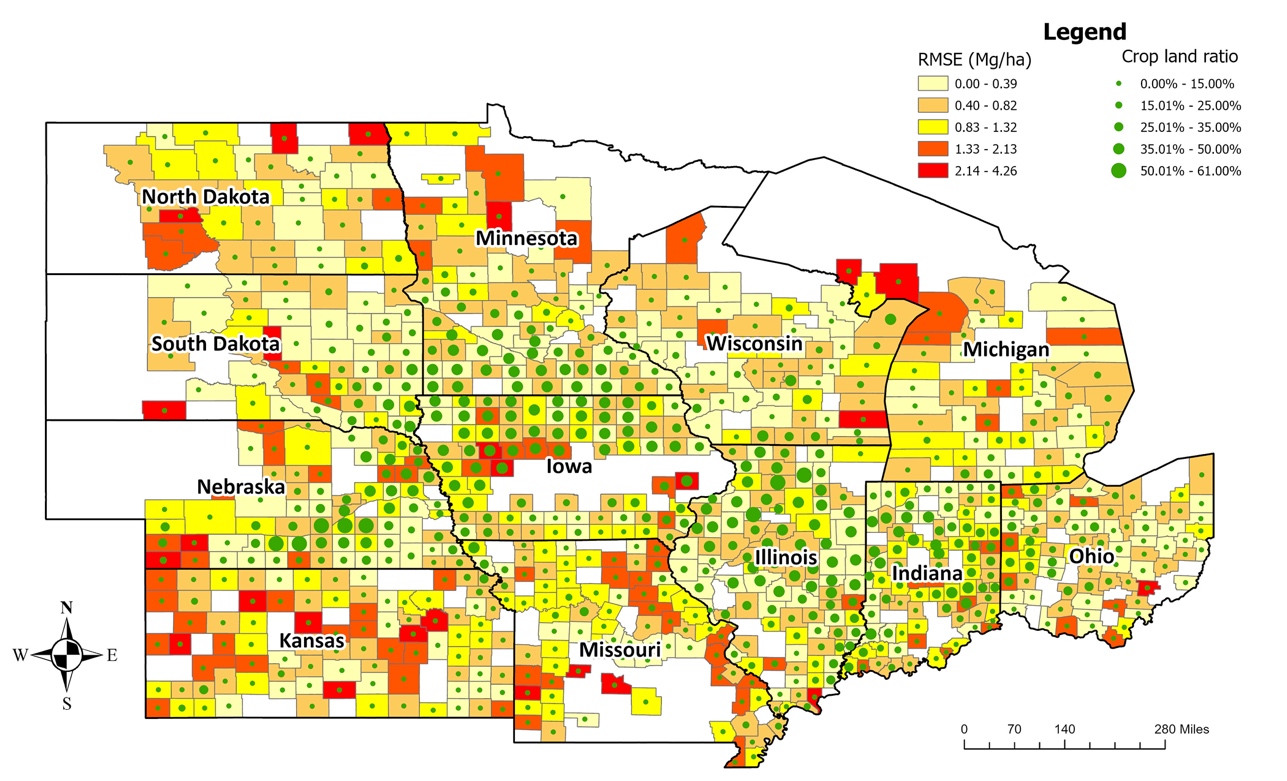
**

**Supplementary Figure 6.** Model performance at the county level in terms of RMSE for the year 2020. The lighter color corresponds to lower RMSE, whereas the darker color stands for higher RMSE. The green circles provide information on the cropland ratio of the county.
